# Nutritional-metabolic indicators do not independently predict AVF thrombosis after adjustment for surgical frequency: a retrospective cohort study

**DOI:** 10.3389/fnut.2026.1845230

**Published:** 2026-07-27

**Authors:** Peng Shu, Zhuping Wen, Yiqi Luo, Xingruo Zeng, Fang Xu

**Affiliations:** The Central Hospital of Wuhan, Huazhong University of Science and Technology, Wuhan, China

**Keywords:** arteriovenous fistula, hypertension, nutritional status, surgical procedures, thrombosis

## Abstract

**Background:**

Arteriovenous fistula (AVF) thrombosis is a leading cause of vascular access failure in hemodialysis patients. Although nutritional status is often hypothesized to influence thrombotic risk, most previous studies have not accounted for surgical burdens such as repeated surgical procedures. This study assessed whether common nutritional and metabolic markers are independently associated with AVF thrombosis after adjustment for surgical frequency.

**Methods:**

This retrospective cohort study included 783 patients who underwent AVF creation between January 2017 and October 2024. Nutritional and metabolic markers were defined using standard formulas. Missing data were handled using multiple imputation. Candidate predictors were identified through univariate analysis (*p* < 0.20). Multivariate logistic regression was initially performed, followed by Cox proportional hazards regression as the primary analysis to properly account for variable follow-up durations. Model discrimination was assessed by the area under the receiver operating characteristic curve (AUC), calibration by the Hosmer-Lemeshow test, and clinical utility by decision curve analysis. Subgroup and sensitivity analyses were performed.

**Results:**

Among 783 patients (median follow-up 34 months), 96 (12.3%) developed AVF thrombosis. Cumulative surgical burden was the strongest independent risk marker (hazard ratio [HR] = 1.44, 95% CI: 1.28–1.62, *p* < 0.001). Hypertension showed a borderline association (HR = 2.24, 95% CI: 0.92–5.43, *p* = 0.080), while diabetes was not independently associated with thrombosis risk (HR = 0.63, 95% CI: 0.38–1.04, *p* = 0.091). No traditional nutritional indices—including prognostic nutritional index (PNI), albumin-to-globulin ratio (AGR), albumin, high-density lipoprotein (HDL), low-density lipoprotein (LDL), vitamin B12, or calcium–phosphate parameters—retained significance after adjustment. The Cox model demonstrated good discriminatory ability (Harrell‘s C-index = 0.74). In complete-case sensitivity analysis (*n* = 165), HDL and vitamin B12 showed associations with thrombosis, though these findings were likely influenced by the substantially reduced sample size and potential selection bias, whereas the primary predictors remained consistent.

**Conclusion:**

After accounting for surgical frequency, conventional nutritional and metabolic indices did not demonstrate independent associations with AVF thrombosis in the present study. Patients with high cumulative surgical burden should be recognized as a high-risk population warranting enhanced surveillance, and optimizing blood pressure control should remain a clinical priority.

## 1 Introduction

Arteriovenous fistula (AVF) remains the preferred vascular access for hemodialysis due to its superior patency and lower complication rates compared to grafts or catheters (1).The pooled 12-month primary patency rate was 73.5% (2), and in a study on arteriovenous fistulas, thrombosis was identified as one of the most common complications, particularly in proximal arteriovenous fistulas where the incidence is higher (3). It leads to dialysis inadequacy, increased hospitalization rates, and substantial healthcare costs. Early identification of patients at high risk for thrombosis is therefore critical to enable timely interventions and preserve access function. Although physical examination and imaging modalities such as ultrasound and angiography are available, they are operator-dependent, invasive, or costly, underscoring the need for simple, non-invasive risk stratification tools (4, 5).

The nutritional and metabolic status has been extensively linked to thrombotic risk. Suboptimal nutritional indicators, including low albumin levels, a decreased prognostic nutritional index (PNI), and an increased neutrophil-to-lymphocyte ratio (NLR), a reduced albumin-to-globulin ratio (AGR) are correlated with endothelial dysfunction, inflammation, and hypercoagulability. These factors may theoretically contribute to the promotion of AVF thrombosis (6–10). Several studies have reported associations between these markers and AVF complications (9–11).

However, most of these investigations did not account for surgical burdens, particularly the number of surgical procedures (e.g., repeated punctures, angioplasties, or surgical revisions), which represent a major source of vascular injury. In a recent machine learning study by our group, the number of surgical procedures was identified as the most important predictor of AVF stenosis (12).While that work highlighted the dominant role of mechanical injury, it did not address whether nutritional indices retain independent predictive value after adjusting for surgical procedures—a critical question for clinical practice.

To fill this gap, the present study was designed to evaluate the independent association of common nutritional and metabolic markers—including the PNI, AGR, vitamin B12, folate, C-reactive protein, and calcium–phosphate parameters—with AVF thrombosis after rigorous adjustment for the number of surgical procedures. We hypothesized that, once the strong confounding effect of mechanical injury is controlled, traditional nutritional indices would not independently predict thrombosis. Using a retrospective cohort of 783 eligible patients after excluding those with missing outcome data, we applied multiple imputation to handle missing data, performed multivariable logistic regression with pooled estimates from five imputed datasets, and conducted extensive sensitivity and subgroup analyses to ensure robustness. Our findings aim to clarify the role of nutritional status in AVF thrombosis and to provide evidence-based guidance for clinical practice.

## 2 Methods

### 2.1 Study design and study population

This was a retrospective cohort study conducted in accordance with the Strengthening the Reporting of Observational Studies in Epidemiology (STROBE) statement (13). Patients who underwent AVF creation or maintenance at the Central Hospital of Wuhan between January 2017 and October 2024 were enrolled.

Inclusion Criteria: 1. Age ≥ 18 years. 2. Underwent AVF creation or revision surgery and used the AVF as the primary permanent vascular access for hemodialysis. 3. Maintenance hemodialysis for ≥ 3 consecutive months before study entry to ensure stable AVF utilization. 4. Availability of baseline clinical, laboratory, and surgical records for analysis.

Exclusion Criteria: 1. Patients with temporary vascular access or using catheters as the main access. 2. Patients with missing values in >30% of core variables or incomplete follow-up records. 3. Patients with severe liver failure, active malignancy, acute infection, or systemic inflammatory disease at baseline, which may severely confound nutritional and inflammatory markers. 4. Patients who received kidney transplantation or died during follow-up before the occurrence of AVF thrombosis. 5. Patients whose outcomes cannot be determined.

A total of 966 patients were initially assessed. After excluding 183 patients with missing outcome data, the final analytical sample comprised 783 patients, of whom 96 (12.3%) developed AVF thrombosis during follow-up. This study was approved by the Ethics Committee of the Central Hospital of Wuhan (Approval No. WHZXKYL2024-115). Written informed consent was waived due to the retrospective, anonymized nature of the study.

Comparison between included and excluded patients. To assess potential selection bias, we compared baseline characteristics between the 783 included patients and the 183 patients excluded due to missing outcome data (Supplementary Table S1).

### 2.2 AVF use duration and post-enrollment follow-up

Follow-up time was defined as the time interval from the date of study enrollment to the earliest occurrence of AVF thrombosis, loss to follow-up, or the last available clinical record. Enrollment occurred after the AVF maturation phase (≥3 months of successful hemodialysis use), and all baseline laboratory measurements were performed at enrollment (Section 2.3). The follow-up period thus represents post-maturation observation time independent of the initial AVF maturation phase, and baseline markers reflect the steady-state nutritional and metabolic status of patients on maintenance hemodialysis.

In addition to post-enrollment follow-up, we also calculated the cumulative duration of AVF use, defined as the time interval from the date of initial AVF creation to the study endpoint (thrombosis, loss to follow-up, or last available record). Based on imputed data (*N* = 783), the median AVF use duration was 34 months (interquartile range [IQR] 43 months), ranging from 1 to 360 months. The maximum value of 360 months (30 years) reflects a patient whose AVF was created long before the study period and remained functional until the endpoint; this is consistent with the study timeline (January 2017 to October 2024), as the actual post-enrollment follow-up for this patient was within the study period.

### 2.3 Outcome and variable definitions

#### 2.3.1 Primary outcome

The primary endpoint was AVF thrombosis, defined as complete occlusion of the AVF confirmed by clinical examination and Doppler ultrasound, requiring interventions such as surgical thrombectomy or percutaneous transluminal angioplasty, or leading to permanent vascular access failure.

#### 2.3.2 Nutritional and metabolic markers

All baseline laboratory measurements were performed at the time of study enrollment, after the AVF had been successfully used for hemodialysis for at least 3 consecutive months. Measurements were not taken at AVF creation or at the initiation of dialysis. For patients with existing AVF at the time of screening, enrollment occurred after the 3-month maturation period, and all laboratory markers were measured within 1 week of enrollment to ensure standardization.

Fistula diameter was measured using Doppler ultrasound at the time of study enrollment, after the AVF maturation phase (≥3 months of successful hemodialysis use).

The following nutritional, metabolic, and inflammatory markers were measured using standard laboratory methods at baseline, with derived indices calculated according to established formulas:

Albumin (g/L) and globulin (g/L), Albumin-to-globulin ratio (AGR) = albumin / globulin.

Prognostic nutritional index (PNI) = albumin (g/L) + 5 × lymphocyte count (×10^9^/L),

Hemoglobin (g/L), Body mass index (BMI) = weight (kg) / height^2^ (m^2^), Neutrophil-to-lymphocyte ratio (NLR) = neutrophil count / lymphocyte count, Ferritin (μg/L), High-sensitivity C-reactive protein (hs-CRP) (mg/L), D-dimer (mg/L), Triglycerides (mmol/L), High-density lipoprotein (HDL) (mmol/L), Low-density lipoprotein (LDL) (mmol/L), Apolipoprotein B / apolipoprotein A1 ratio (ApoB/ApoA1), Vitamin B12 (pmol/L), Folate (nmol/L), Calcium (mmol/L) and phosphorus (mmol/L).

Calcium–phosphate product = calcium × phosphorus.

Covariates: age, sex, smoking status, alcohol consumption, hypertension, diabetes mellitus, surgical history: surgery-count, fistula_diameter, Follow-up time.

Serum albumin was measured using the bromocresol green (BCG) method, with a normal reference range of 35–55 g/L in our laboratory. Blood samples were collected prior to dialysis sessions.

Surgery count was defined as the total number of all surgical and endovascular interventions performed on the same arteriovenous fistula access site prior to study enrollment. This included: (a) initial AVF creation surgery; (b) percutaneous transluminal angioplasty (PTA) for stenosis; (c) surgical thrombectomy; (d) AVF revision or repair; and (e) other interventional procedures performed on the AVF (e.g., angioplasty with stenting, or fistulography with subsequent intervention). Diagnostic fistulography or angiography without intervention was not counted. Each procedure was counted as a separate event; for patients who underwent multiple interventions during a single session, each was counted individually. The time window for counting was from the date of initial AVF creation to the date of study enrollment. Procedures performed after enrollment were not included in the baseline surgery count variable.

### 2.4 Data preprocessing and missing data handling

Multiple imputation by chained equations (MICE) was performed using predictive mean matching (PMM) to generate 50 imputed datasets with 20 iterations, including all analysis variables. The imputation procedure was performed under the missing at random (MAR) assumption, which assumes that missingness depends only on observed variables and not on the unobserved values themselves. To assess the plausibility of this assumption, we performed a missing indicator method: missing indicators were created for all variables with >30% missingness and included in the logistic model alongside imputed values. None of the missing indicators were significantly associated with thrombosis (all *p* > 0.05), supporting the MAR assumption.

To further validate the robustness of our findings, four sensitivity analyses were performed:

1. Complete-case analysis: restricting to patients with complete data on all analysis variables (*n* = 165);
2. Median imputation: replacing missing values with the median of observed values for each variable;
3. Missing indicator method: including missing indicators for variables with >30% missingness in the model alongside imputed values (all missing indicators *p* > 0.05);
4. Best- and worst-case scenario imputation: for the primary exposure (surgery count), assigning all missing values to the lowest observed value (best-case) or the highest observed value (worst-case). Best-case: OR = 2.05; worst-case: OR = 0.91. To assess the plausibility of this assumption, we tested whether missingness for each variable was associated with the outcome (thrombosis) using logistic regression; no significant associations were found (all *p* > 0.05; Supplementary Table S2), supporting the MAR assumption.

To evaluate the missing data mechanism, we compared baseline characteristics between patients with observed versus missing values for each variable with >30% missingness using the Mann–Whitney U test for continuous variables and the chi-square test for categorical variables (Supplementary Table S5). For surgery count, the primary exposure variable, no significant differences were observed between observed and missing groups across all baseline characteristics (all *p* > 0.05), supporting the MAR assumption. For HDL and LDL, missingness was associated with albumin, BMI, and diabetes, suggesting that missingness depended on observed characteristics, which is consistent with MAR. For the primary exposure (surgery count), we additionally performed extreme-case imputations (best- and worst-case scenarios) as a sensitivity analysis under a plausible MNAR scenario.

### 2.5 Baseline characteristics

Baseline characteristics are presented using pooled estimates derived from the 50 imputed datasets, with *p*-values combined using Rubin‘s rules (D2 method). Continuous variables are presented as median (IQR), and categorical variables as n (%). Between-group comparisons were performed using the Wilcoxon rank-sum test for continuous variables and the chi-square test for categorical variables, with *p*-values pooled across imputations.

### 2.6 Statistical analysis

Univariate logistic regression models were constructed to examine the crude association between each candidate variable and the risk of AVF thrombosis. Variables with a *p* value < 0.20 in univariate analysis were retained for inclusion in the multivariate model to avoid exclusion of potential confounders. To properly handle the time-to-event nature of AVF thrombosis, we performed Cox proportional hazards regression as the primary analytical approach. The proportional hazards assumption was verified using Schoenfeld residual tests and was satisfied for all primary exposure variables (surgery_count: *p* = 0.380; hypertension: *p* = 0.906; diabetes: *p* = 0.206). Harrell‘s C-index was calculated to assess model discrimination. Given the variable follow-up duration (ranging from 1 to 360 months), the Cox model provides more accurate risk estimates than logistic regression by appropriately censoring patients at their last known follow-up time. Surgery count was entered into the Cox model as a baseline fixed covariate (cumulative count prior to enrollment), reflecting the total vascular injury burden before nutritional-metabolic marker assessment.

Multicollinearity among independent variables was evaluated using the variance inflation factor (VIF). A VIF value < 5 was defined as the threshold for absence of severe multicollinearity, ensuring stability of the regression coefficients.

The performance of the final multivariate model was assessed using multiple metrics. Discriminatory ability was quantified using the area under the receiver operating characteristic curve (AUC) with 95% CI. Calibration was evaluated using the Hosmer–Lemeshow goodness-of-fit test and the Brier score. Clinical utility was further examined using decision curve analysis (DCA) to quantify the net benefit of the model across threshold probabilities.

To explore the consistency of associations, stratified subgroup analyses were conducted according to age (< 60 vs. ≥ 60 years) and sex. Sensitivity analyses were performed using complete-case analysis, median imputation, and data trimming after exclusion of extreme values to verify the robustness of the primary findings.

Internal validation was performed using bootstrap resampling with 500 iterations to assess model stability and optimism. For the logistic regression model, we calculated the optimism-corrected area under the ROC curve (AUC) and calibration slope. For the Cox regression model, we calculated the optimism-corrected Harrell’s C-index. Optimism was defined as the difference between the apparent performance (measured in the original dataset) and the average performance across bootstrap samples. Small optimism values indicate minimal overfitting and good model generalizability.

The events-per-variable (EPV) ratio for the final Cox model was calculated as the number of thrombosis events (*n* = 96) divided by the number of predictor variables included in the model (*n* = 5), yielding an EPV of 19.2, which exceeds the traditionally recommended minimum of 10 and supports the stability of the regression estimates.

To assess the linearity assumption for surgery count, we performed restricted cubic spline (RCS) regression with 3 knots in the logistic model, with a test for nonlinearity. As a sensitivity analysis, we also analyzed surgery count as a categorical variable (0, 1, 2, ≥3; Supplementary Table S6) to assess dose–response patterns and potential threshold effects.

With 96 thrombosis events and 5 predictors in the final Cox model, the study had approximately 80% power to detect a hazard ratio of 1.6 or greater for continuous predictors at *α* = 0.05. However, the study was not specifically powered to detect modest independent effects of nutritional-metabolic indicators, and the wide confidence intervals observed for markers such as vitamin B12 and HDL suggest that smaller but potentially clinically meaningful associations cannot be excluded.

### 2.7 Software

All statistical analyses and data visualizations were performed using R software (version 4.4.2). The following packages were used for data management, imputation, regression modeling, and graphical presentation: tidyverse, mice, rms, pROC, tableone, car, forestplot, and ResourceSelection.

## 3 Results

**Figure 1:**
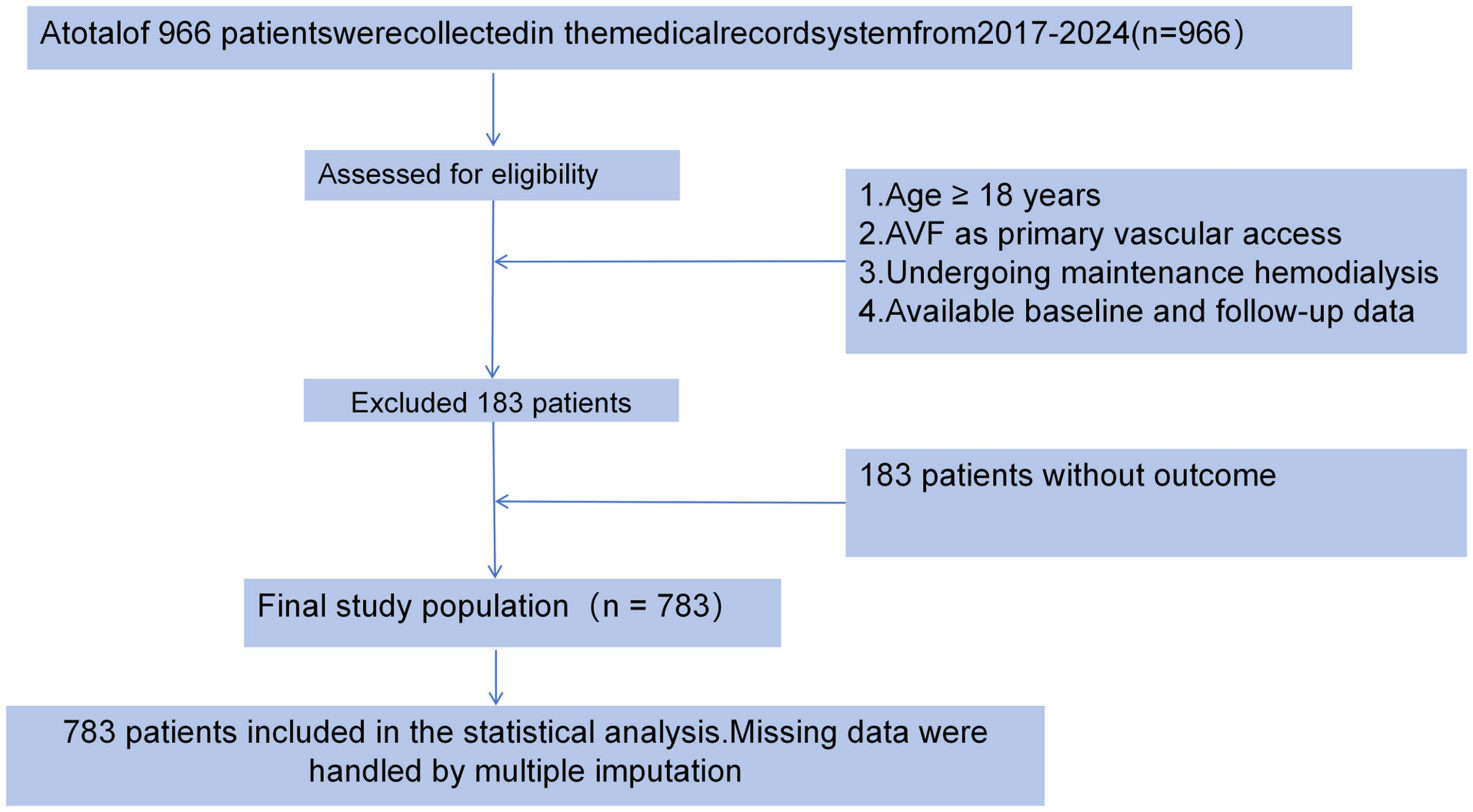
Flow diagram of patient enrollment, eligibility assessment, and analysis.

The flow diagram detailing patient enrollment and eligibility assessment is displayed in Figure 1.

### 3.1 Baseline characteristics of study population

Patients who developed thrombosis had significantly higher levels of vitamin B12 (*p* < 0.001) and a greater number of previous vascular access surgeries (*p* < 0.001) compared with those without thrombosis. AVF use duration was also longer in the thrombosis group (*p* = 0.066). D-dimer levels were lower in the thrombosis group (*p* = 0.062; Table 1). Surgery count was calculated cumulatively from the date of initial AVF creation to the date of study enrollment, including all surgical and endovascular interventions performed on the same vascular access site prior to baseline. To assess potential selection bias, we compared baseline characteristics between the 783 included patients and the 183 patients excluded due to missing outcome data (Supplementary Table S1). No statistically significant differences were observed between the two groups in age (*p* = 0.303), gender (*p* = 0.793), hypertension (*p* = 0.247), diabetes (*p* = 0.839), surgery count (*p* = 0.409), albumin (*p* = 0.275), hemoglobin (*p* = 0.619), or BMI (*p* = 0.589). These findings suggest that exclusion due to missing outcome data did not introduce substantial systematic bias with respect to measured baseline characteristics.

**Table 1:** Baseline demographic, clinical, laboratory, and surgical characteristics of the study population according to arteriovenous fistula thrombosis status.

Variable	No thrombosis (*n* = 687)	Thrombosis (*n* = 96)	*p*
Albumin (g/L), median (IQR)	65.80 (59.75–72.40)	66.90 (60.03–72.45)	0.461
AGR, median (IQR)	2.25 (2.07–2.43)	2.20 (2.08–2.46)	0.568
PNI, median (IQR)	71.45 (65.00–78.90)	72.70 (64.46–79.94)	0.449
Hemoglobin (g/L), median (IQR)	90.00 (76.00–107.00)	92.00 (81.00–108.25)	0.263
BMI (kg/m^2^), median (IQR)	23.40 (21.10–25.30)	22.50 (20.60–25.42)	0.253
NLR, median (IQR)	3.87 (2.80–5.89)	3.36 (2.60–4.88)	0.073
Ferritin (μg/L), median (IQR)	124.43 (51.80–259.73)	89.16 (42.71–255.99)	0.471
hs-CRP (mg/L), median (IQR)	1.21 (0.29–4.43)	1.62 (0.38–5.52)	0.312
D-dimer (mg/L), median (IQR)	0.95 (0.49–1.79)	0.88 (0.44–1.33)	0.138
Triglycerides (mmol/L), median (IQR)	1.34 (0.85–1.98)	1.20 (0.94–1.91)	0.381
HDL (mmol/L), median (IQR)	1.02 (0.83–1.24)	1.08 (0.89–1.31)	0.152
LDL (mmol/L), median (IQR)	2.26 (1.67–2.87)	2.34 (1.85–3.11)	0.253
ApoB/ApoA1 ratio, median (IQR)	0.72 (0.60–0.94)	0.70 (0.56–0.95)	0.456
Vitamin B12 (pmol/L), median (IQR)	275.00 (201.00–416.50)	346.50 (219.00–499.00)	**< 0.001**
Folate (nmol/L), median (IQR)	17.00 (11.15–28.23)	18.20 (10.67–30.66)	0.477
Calcium (mmol/L), median (IQR)	2.18 (2.01–2.34)	2.20 (2.02–2.39)	0.361
Phosphorus (mmol/L), median (IQR)	1.59 (1.29–1.91)	1.62 (1.40–1.97)	0.371
Ca × P product (mmol^2^/L^2^), median (IQR)	3.42 (2.76–4.21)	3.58 (2.88–4.14)	0.245
Age (years), median (IQR)	62.00 (52.50–69.00)	62.00 (54.00–70.00)	0.455
Gender (Male), n (%)	474 (69.0)	60 (62.5)	0.225
Smoking, n (%)	142 (20.7)	25 (26.0)	0.253
Alcohol consumption, n (%)	66 (9.6)	11 (11.5)	0.699
Diabetes, n (%)	463 (67.4)	55 (57.3)	0.056
Hypertension, n (%)	603 (87.8)	90 (93.8)	0.096
Surgery count categories, n (%)			**< 0.001**
0	373 (44.4)	9 (7.2)	
1	331 (39.4)	55 (44.0)	
2	90 (10.7)	29 (23.2)	
≥3	47 (5.6)	32 (25.6)	
Fistula diameter (cm), median (IQR)	0.41 (0.30–0.53)	0.42 (0.32–0.53)	0.190
AVF use duration (months), median (IQR)	33.00 (17.00–59.50)	43.50 (17.75–68.00)	0.053

Data are based on 50 imputed datasets. *p*-values were pooled across imputations using Rubin’s rules (D2 method). Continuous variables are presented as median (IQR) and categorical variables as n (%). Bold values indicate statistically significant between-group differences for the corresponding variable.g variable.

Although NLR showed a borderline difference in unadjusted between-group comparison (*p* = 0.073), this finding should be interpreted with caution, as the association was not significant in univariate logistic regression (*p* = 0.329), likely reflecting the influence of confounding factors such as follow-up time.

### 3.2 Missing data and multicollinearity assessment

The distribution of missing values for each variable is presented in Table 2. Relatively high missing proportions were observed for vitamin B12 (59.5%), folate (56.7%), hs-CRP (42.3%), surgery count (39.1%), and ferritin (38.3%).

**Table 2:** Missing data distribution for all variables.

Variable	Missing_N	Missing_Percent
Thrombosis	183	18.94
Albumin	70	7.25
AGR	71	7.35
PNI	77	7.97
Hemoglobin	40	4.14
BMI	220	22.77
NLR	37	3.83
ferritin	370	38.3
Hs-crp	409	42.34
D-dimer	298	30.85
Triglycerides	302	31.26
HDL	328	33.95
LDL	330	34.16
ApoB/ApoA1	327	33.85
Vitamin B12	575	59.52
Folate	548	56.73
Calcium	41	4.24
Phosphorus	54	5.59
Ca × P product	60	6.21
Age	26	2.69
Gender	2	0.21
Smoking	23	2.38
Alcohol	24	2.48
Diabetes	0	0
Hypertension	0	0
Surgery count	378	39.13
Fistula diameter	307	31.78
Time	140	14.49

**Figure 2:**
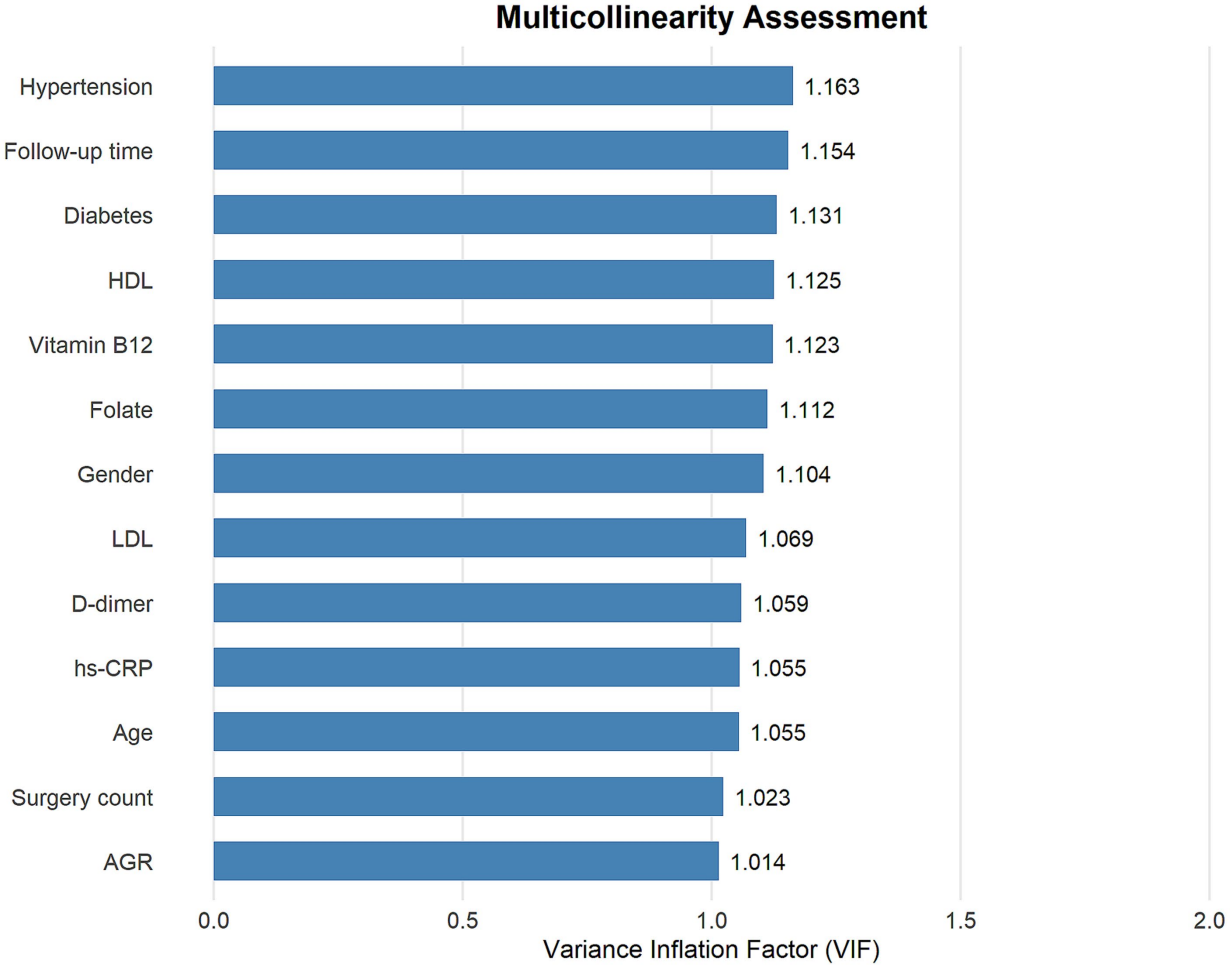
Variance inflation factor (VIF) of the candidate variables.

Multicollinearity was assessed using the variance inflation factor (VIF) for all variables entered into the multivariate model. As shown in Figure 2, all VIF values were well below the commonly accepted threshold of 5, with the highest value observed at approximately 1.8. These findings confirmed the absence of severe multicollinearity and supported the stability of the regression estimates.

Sensitivity analyses across different missing data approaches consistently identified surgery count as the dominant risk marker. In the complete-case analysis (*n* = 165), surgery count remained a significant predictor (HR = 1.47, 95% CI: 1.07–2.10, *p* = 0.021). The missing indicator method showed no significant association between missingness indicators and thrombosis (all *p* > 0.05; Supplementary Table S2), suggesting that missingness was not informative. In the best/worst-case imputations for surgery count, the association remained consistently significant (*p* < 0.05) in both extreme scenarios, further supporting the robustness of our primary findings.

Comparisons between patients with observed versus missing values for each high-missing variable are presented in Supplementary Table S5. For surgery count, the primary exposure variable, no significant differences were observed between observed and missing groups in age (*p* = 0.888), albumin (*p* = 0.865), hemoglobin (*p* = 0.465), BMI (*p* = 0.848), gender (*p* = 0.394), hypertension (*p* = 0.331), or diabetes (*p* = 0.863). For vitamin B12 and folate, missingness was marginally associated with albumin levels (*p* = 0.036 and *p* = 0.009, respectively), with no other significant differences. For HDL and LDL, missingness was associated with albumin, BMI, and diabetes, suggesting that missingness depended on observed characteristics, consistent with MAR.

### 3.3 Univariate logistic regression analysis

Univariate logistic regression was conducted to screen potential risk factors for AVF thrombosis. Variables with *p* < 0.20 were entered into the subsequent multivariate Cox regression model. As shown in Table 3, surgery count, HDL, vitamin B12, follow-up time, D-dimer, diabetes, folate, hypertension, hs-CRP, and LDL were candidate variables for multivariate analysis (all *p* < 0.20). Among these, surgery count (odds ratio [OR] = 1.70, *p* < 0.001), HDL (OR = 2.27, *p* = 0.002), vitamin B12 (OR = 1.001, *p* = 0.002), and follow-up time (OR = 1.006, *p* = 0.013) were significantly associated with an increased risk of AVF thrombosis, while D-dimer showed a protective association (OR = 0.85, *p* = 0.045).

**Table 3:** Univariate analysis for screening candidate predictors of AVF thrombosis.

Variable	OR	Lower	Upper	*p*
Surgery_count	1.697	1.443	1.996	<0.001
HDL	2.267	1.344	3.826	0.002
Vitb12	1.001	1.000	1.002	0.002
Time	1.006	1.001	1.011	0.013
D_dimer	0.850	0.725	0.996	0.045
Diabetes	0.649	0.420	1.002	0.051
Folate	1.002	1.000	1.005	0.077
Hypertension	2.090	0.887	4.925	0.092
hs_crp	1.005	0.999	1.011	0.133
LDL	1.157	0.940	1.424	0.169
Gender	0.749	0.481	1.167	0.202
Smoking	1.351	0.826	2.210	0.230
Fistula_diameter	1.622	0.655	4.018	0.296
NLR	0.979	0.939	1.021	0.329
Albumin	1.008	0.986	1.031	0.479
Triglycerides	0.934	0.770	1.133	0.489
Alcohol	1.218	0.619	2.397	0.569
Hemoglobin	1.002	0.995	1.009	0.588
Ferritin	1.000	0.999	1.000	0.601
ApoB_ApoA1	1.000	0.999	1.002	0.622
PNI	1.004	0.987	1.021	0.646
Phosphorus	0.967	0.819	1.142	0.692
BMI	0.990	0.935	1.048	0.723
Age	1.003	0.986	1.019	0.761
Ca_p_product	0.991	0.934	1.051	0.763
AGR	0.954	0.491	1.852	0.889
Calcium	0.996	0.729	1.362	0.982

Notably, although NLR showed a borderline difference in unadjusted between-group comparison (Table 1: *p* = 0.073), it was not significantly associated with thrombosis in univariate logistic regression (*p* = 0.329). This discrepancy likely reflects the influence of confounding factors such as follow-up time, as patients who developed thrombosis had longer follow-up durations.

### 3.4 Multivariate cox regression analysis

**Figure 3:**
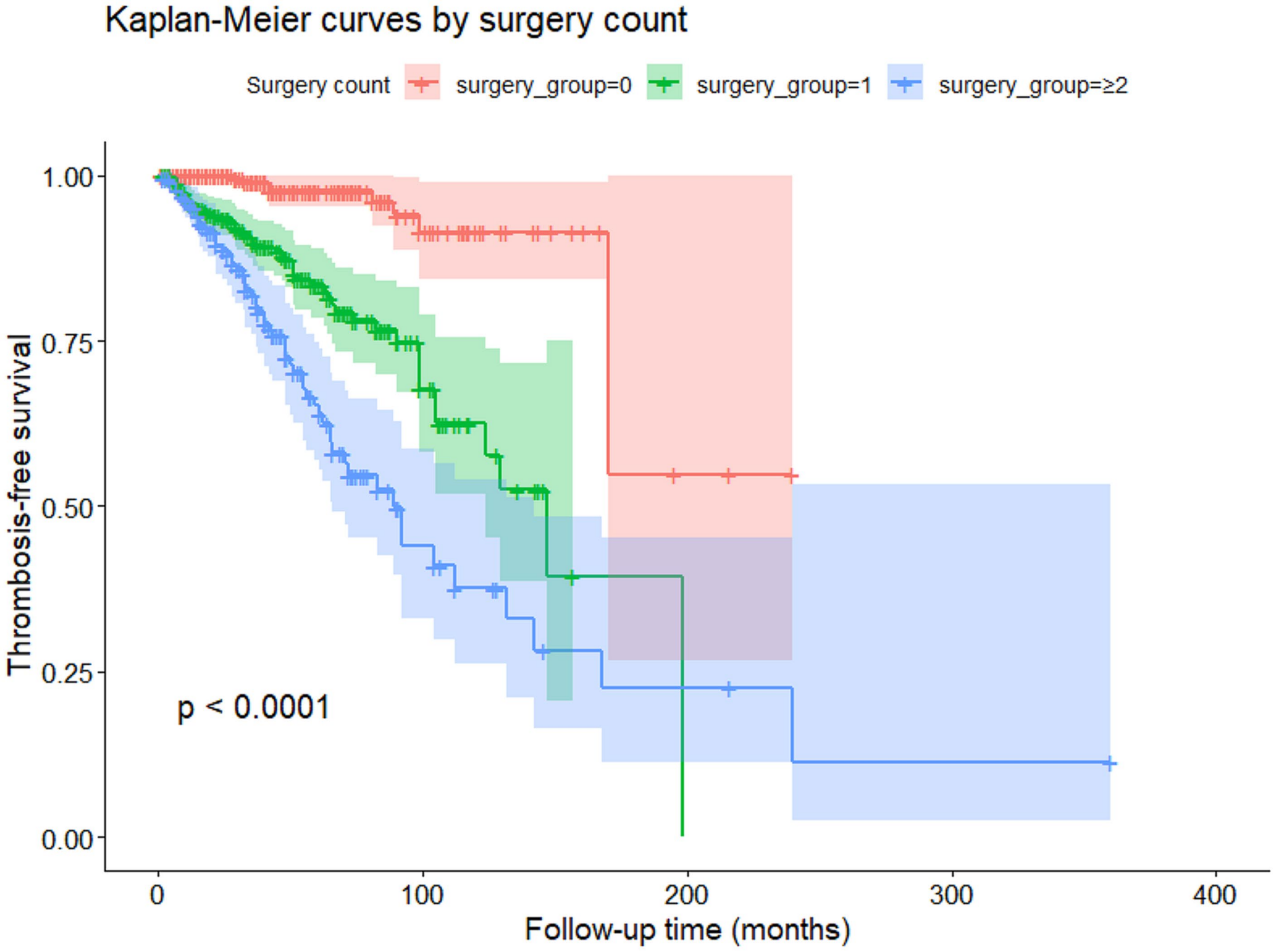
Kaplan–Meier curves for thrombosis-free survival stratified by surgery count groups (0, 1, and ≥2 procedures). The log-rank test showed a significant difference between groups (*p* < 0.001).

Cox proportional hazards regression was performed to identify independent predictors of AVF thrombosis while accounting for variable follow-up times. After adjusting for surgery count, hypertension, diabetes, age, and gender, the number of surgical procedures remained the strongest independent risk factor (HR = 1.44, 95% CI: 1.28–1.62, *p* < 0.001). Hypertension showed a borderline association (HR = 2.24, 95% CI: 0.92–5.43, *p* = 0.080), while diabetes was not independently associated with thrombosis risk (HR = 0.63, 95% CI: 0.38–1.04, *p* = 0.091). Age and gender were not significant predictors (*p* = 0.063 and *p* = 0.946, respectively). The proportional hazards assumption was satisfied for the core predictors of interest (surgery_count, hypertension, and diabetes; all *p* > 0.05), and the model demonstrated good discriminatory ability (Harrell‘s C-index = 0.74). The Kaplan–Meier curve (Figure 3) showed significantly lower thrombosis-free survival in patients with higher surgical burden (log-rank *p* < 0.001). Other variables, including vitamin B12, HDL, hs-CRP, folate, LDL, D-dimer, and follow-up time, did not reach statistical significance after adjustment in this cohort (all *p* > 0.05).

The final Cox model included 5 core predictors (surgery count, hypertension, diabetes, age, and gender), with an events-per-variable (EPV) ratio of 19.2 (96 events / 5 predictors), exceeding the recommended minimum of 10.

The linearity assumption for surgery count was assessed using restricted cubic spline regression, which showed no significant nonlinearity (*p* = 1.000), supporting the use of surgery count as a continuous predictor in the primary Cox model. When analyzed categorically (0, 1, 2, ≥3), surgery count showed a stepwise increase in thrombosis risk: HRs were 5.47 (95% CI: 2.39–12.49) for 1 procedure, 8.71 (95% CI: 3.77–20.14) for 2 procedures, and 13.06 (95% CI: 4.61–36.96) for ≥3 procedures compared with 0 procedures (P for trend < 0.001; Supplementary Table S6), consistent with a linear dose–response relationship.

Strata-specific thrombosis events. The distribution of AVF thrombosis events across key clinical strata was as follows. Among patients with diabetes, 55 of 518 (10.6%) developed thrombosis, compared with 41 of 265 (15.5%) among those without diabetes. Among patients with hypertension, 90 of 693 (13.0%) developed thrombosis, compared with 6 of 90 (6.7%) among those without hypertension. By age, 60 of 451 (13.3%) patients aged ≥60 years developed thrombosis, compared with 35 of 323 (10.8%) among those aged <60 years. By sex, 60 of 534 (11.2%) males and 36 of 249 (14.5%) females developed thrombosis.

### 3.5 Subgroup analysis

**Figure 4:**
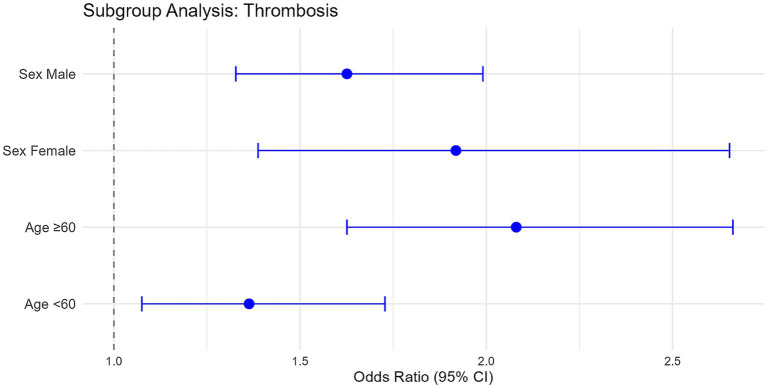
Forest plot of subgroup analyses stratified by age and sex.

Stratified subgroup analyses were performed according to age (<60 vs. ≥60 years) and sex to evaluate the consistency of the primary association across different populations. The results are visualized in Figure 4. The association between surgery count and AVF thrombosis was significant in all subgroups. The odds ratios (95% CIs) were 1.36 (1.08–1.73) in patients aged <60 years and 2.08 (1.63–2.66) in those aged ≥60 years; for sex, the ORs were 1.63 (1.33–1.99) in males and 1.92 (1.39–2.65) in females. No significant interaction was detected (P for interaction > 0.05 for both age and sex), indicating that the effect of surgery count did not differ significantly across these subgroups.

### 3.6 Model performance

The discrimination and clinical utility of the predictive model were comprehensively evaluated using Harrell’s C-index and decision curve analysis (DCA).

**Figure 5:**
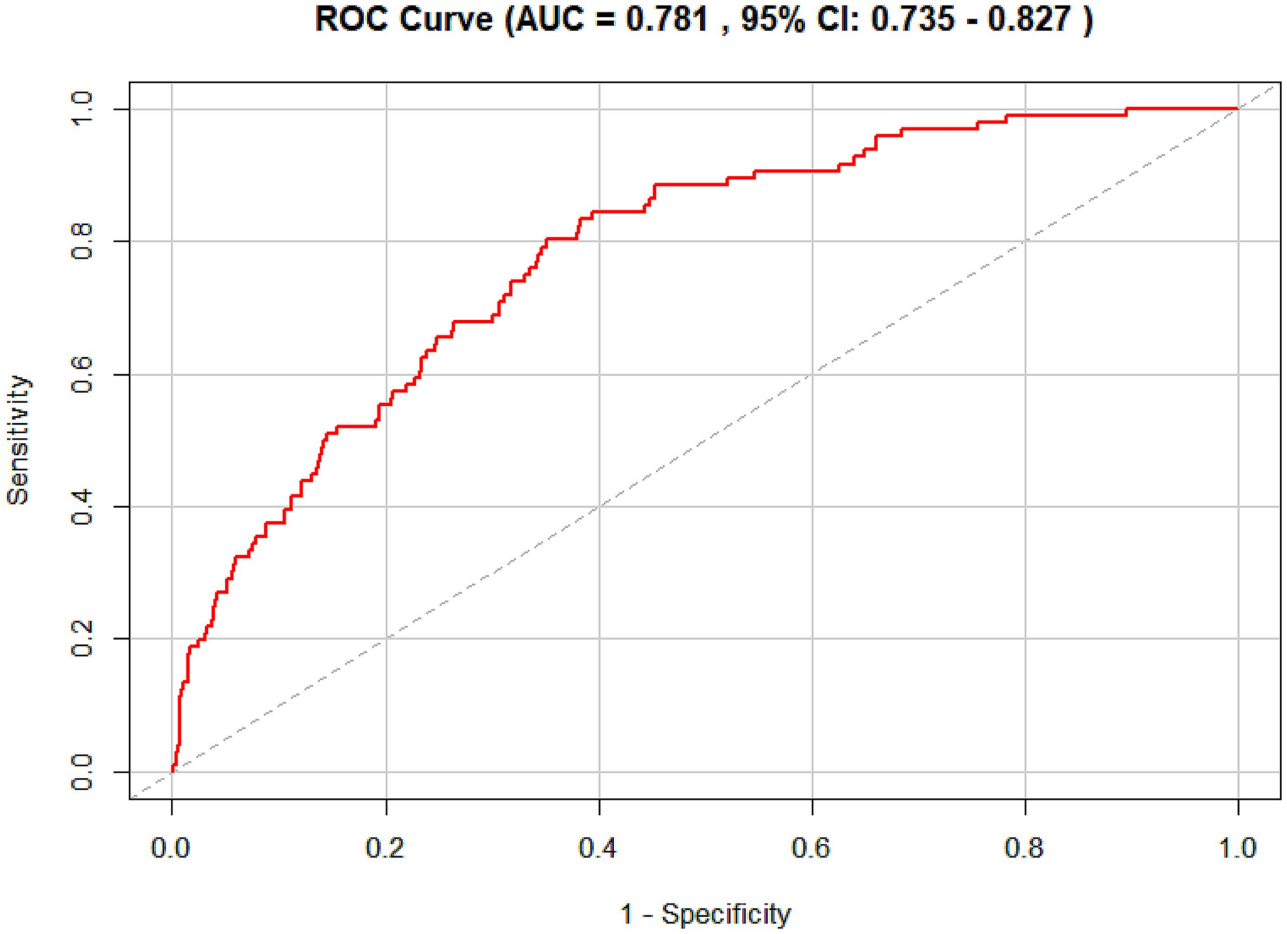
Receiver operating characteristic (ROC) curve of the predictive model for AVF thrombosis.

The receiver operating characteristic (ROC) curve yielded an area under the curve (AUC) of 0.781 (0.735–0.827), indicating good discriminative ability for identifying patients at high risk of AVF thrombosis (Figure 5).

**Figure 6:**
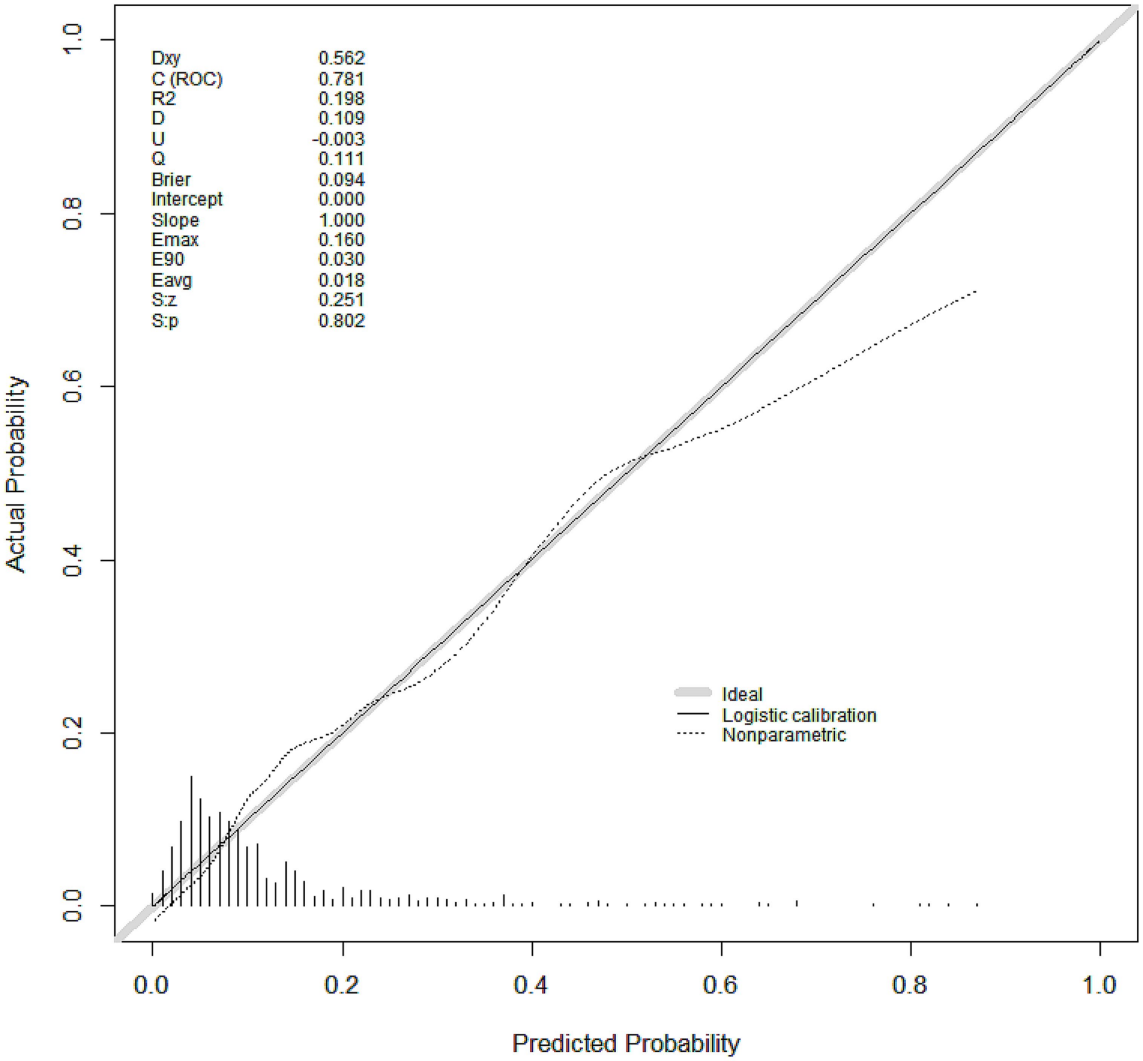
Calibration curve of the predictive model for AVF thrombosis.

The calibration curve showed good agreement between the model’s predicted probabilities and the observed outcomes, with the logistic calibration curve lying close to the ideal 45 diagonal line (Figure 6). The Hosmer-Lemeshow test yielded a *p* value of 0.591, indicating no significant lack of fit, and the Brier score was 0.094, reflecting favorable overall predictive accuracy.

**Figure 7:**
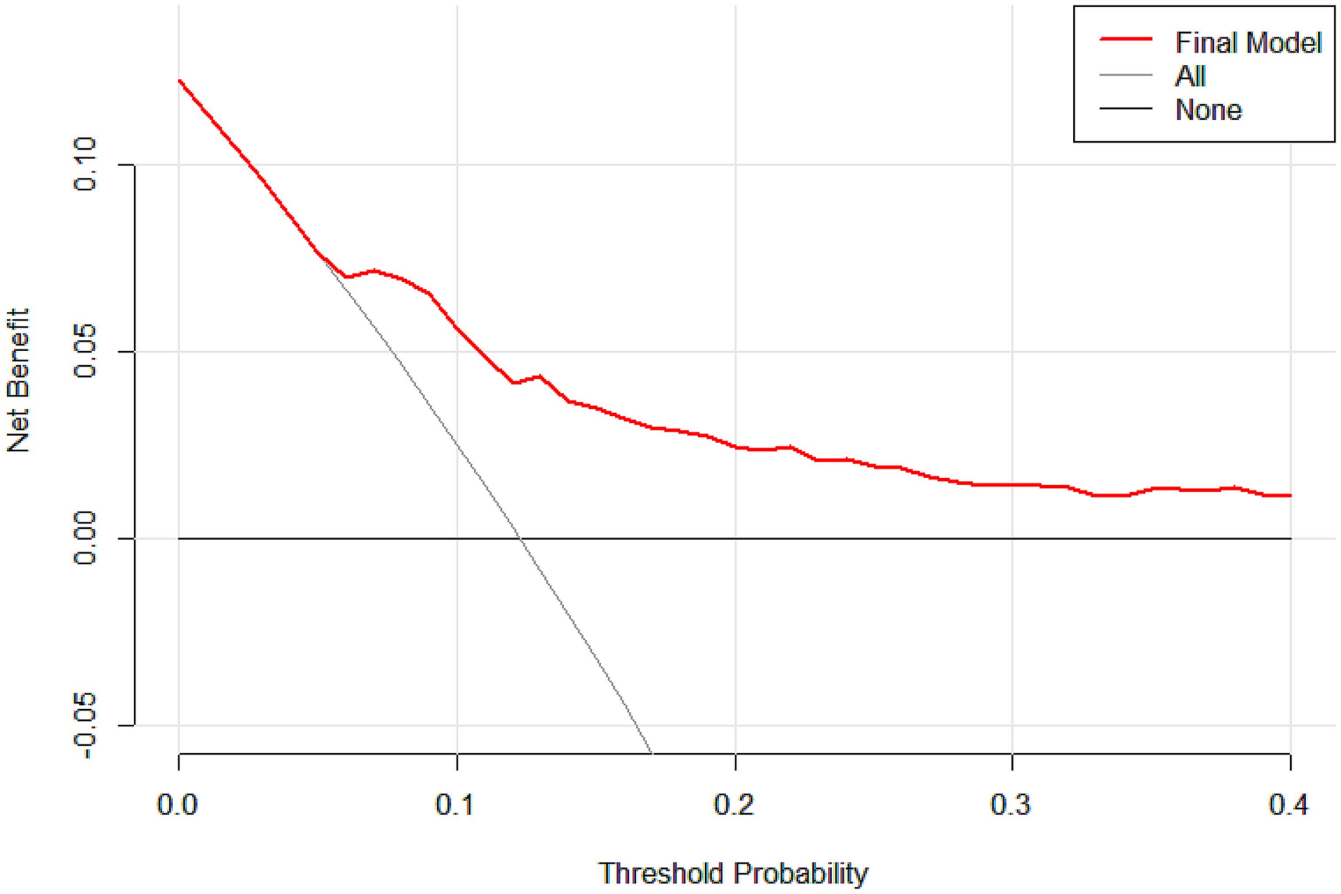
Decision curve analysis (DCA) showing the net benefit of the predictive model.

Decision curve analysis (DCA) was used to evaluate the clinical net benefit of the model. As shown in Figure 7, the predictive model provided positive net benefit across threshold probabilities ranging from 0 to 0.40, consistently outperforming the “treat all” and “treat none” strategies. These findings support the potential clinical utility of the model for risk stratification of AVF thrombosis.

Internal validation using bootstrap resampling with 500 iterations confirmed the stability of both models. For the logistic model, the apparent AUC was 0.845, with a bootstrap mean of 0.850 and an optimism of 0.0053, yielding an optimism-corrected AUC of 0.840. The optimism-corrected calibration slope was 6.057 (apparent: 6.132). For the Cox model, the apparent C-index was 0.740, with a bootstrap mean of 0.746 and an optimism of 0.0065, yielding an optimism-corrected C-index of 0.733. These extremely low optimism values suggest minimal overfitting and good model generalizability (Supplementary Table S4).

### 3.7 Sensitivity analysis

Sensitivity analyses were performed to assess the robustness of the primary findings. Complete-case analysis (*n* = 165) showed surgery count remained a significant independent risk marker (HR = 1.47, *p* = 0.021). Median imputation (*n* = 783) produced an AUC of 0.770, consistent with the primary model. The missing indicator method showed that missingness indicators for all high-missing variables were not significantly associated with thrombosis (all *p* > 0.05, ranging from 0.092 for surgery_count_miss to 0.982 for ldl_miss), confirming that missingness was not informative. Best- and worst-case imputation for surgery count demonstrated that under the best-case scenario (missing assigned to minimum value), surgery count remained a significant risk factor (OR = 2.05); under the worst-case scenario (missing assigned to maximum value), the association was attenuated (OR = 0.91), which is expected given the extreme and clinically implausible assumption. Taken together, these findings support the robustness of the primary results derived from multiple imputation. The missing indicator method showed that missingness indicators for all high-missing variables were not significantly associated with thrombosis (all *p* > 0.05; Supplementary Table S3), confirming that missingness was not informative.

Additionally, internal validation through bootstrap resampling (500 iterations) demonstrated excellent model stability, with minimal optimism for both logistic (optimism = 0.0053; optimism-corrected AUC = 0.840) and Cox (optimism = 0.0065; optimism-corrected C-index = 0.733) models, further supporting the robustness of our findings (Supplementary Table S4).

In complete-case analysis (*n* = 165), surgery count remained a significant independent risk marker (HR = 1.47, 95% CI: 1.07–2.10, *p* = 0.021). However, in this reduced sample, HDL and vitamin B12 also showed significant associations with thrombosis (Supplementary Table S1). This divergence is likely attributable to the substantially reduced sample size (*n* = 165, representing only 21.1% of the original cohort) and potential selection bias, as patients with complete data may differ systematically from those with missing values. Importantly, the direction and significance of the primary predictors (surgery count and hypertension) remained consistent across all analytical approaches, supporting the robustness of our conclusions (Table 4).

**Table 4:** Cox proportional hazards regression analysis for AVF thrombosis.

Variable	HR (95% CI)	*p*-value	PH assumption *p*-value
Surgery count	**1.44 (1.28–1.62)**	**< 0.001**	0.380
Hypertension	2.24 (0.93–5.43)	0.080	0.906
Diabetes	0.63 (0.38–1.04)	0.091	0.206
Age (years)	1.02 (1.00–1.04)	0.063	0.016
Gender (Male vs. Female)	1.02 (0.65–1.60)	0.946	0.021

Bold values indicate statistically significant between-group differences for the corresponding variable.g variable.

## 4 Discussion

In the present retrospective cohort study including 783 hemodialysis patients with arteriovenous fistula, we investigated the independent associations between nutritional-metabolic indicators and the risk of AVF thrombosis, with deliberate adjustment for the number of surgical procedures—a major mechanical confounder that has been largely overlooked in previous studies. Our key finding was that surgical frequency and hypertension remained robust independent risk factors for AVF thrombosis, whereas conventional nutritional and metabolic markers showed no independent predictive value after full multivariable adjustment. These results support our hypothesis that, once the substantial confounding effect of repeated surgical injury is accounted for, classic nutritional biomarkers do not contribute independently to the pathogenesis of AVF thrombosis. The robustness of these findings was further confirmed through multiple sensitivity analyses, all of which consistently identified surgical burden as the dominant risk factor.

Importantly, the observed association between surgery count and AVF thrombosis should be interpreted with caution regarding causality. As noted above, surgery count may serve as a proxy for underlying vascular disease severity rather than a purely causal exposure. Specifically, each surgical procedure is typically performed because the fistula is already failing (e.g., due to progressive stenosis or reduced flow), which means that confounding by indication is inherent to this analysis—patients with more severe vascular pathology receive more interventions, and these same patients are also at higher risk of thrombosis. Furthermore, reverse causation cannot be fully excluded: repeated procedures indicate progressive vascular pathology, which itself drives thrombosis risk. Thus, surgery count is best interpreted as a composite risk marker that captures both the cumulative procedural burden and the severity of the underlying vascular disease, rather than a direct causal agent. This distinction is essential for correct clinical interpretation: patients with elevated surgery counts should be recognized as a high-risk population warranting intensified surveillance, rather than implying that all prior procedures were unnecessary or avoidable.

A growing body of evidence has linked nutritional disturbances, metabolic disorders, and chronic inflammation to adverse outcomes in hemodialysis patients (14). Malnutrition-inflammation complex syndrome is known to promote endothelial dysfunction, vascular remodeling, and a prothrombotic state, all of which might predispose patients to vascular access failure (15, 16). Accordingly, numerous studies have suggested that biomarkers such as vitamin B12, folate, lipid profiles, and inflammatory markers may predict AVF dysfunction (17–19). In our unadjusted analyses, several nutritional-related indicators, including vitamin B12 and HDL, were significantly associated with AVF thrombosis, consistent with these previous observations.

However, these associations were no longer significant after multivariable adjustment that included surgery count. Specifically, vitamin B12, HDL, and other nutritional-metabolic indicators showed no independent association with AVF thrombosis in the fully adjusted model. This finding suggests that nutritional-metabolic indicators may not act as independent causal factors, but rather serve as surrogate markers of overall disease severity, repeated vascular injury, or sustained inflammation. Most prior studies investigating nutritional biomarkers failed to incorporate the number of surgical procedures into their analytical models (18, 20). Because repeated interventions directly cause vascular damage, intimal hyperplasia, and flow disturbance, they represent a far more dominant determinant of AVF thrombosis than systemic metabolic status alone. Our study therefore provides a more evaluation by isolating the true independent effect of nutrition after accounting for this critical mechanical confounder.

Importantly, the absence of statistically significant associations for nutritional-metabolic indicators such as vitamin B12 and HDL in the fully adjusted model should not be interpreted as definitive evidence that these markers have no predictive value. As the reviewer correctly noted, absence of evidence is not evidence of absence. The wide confidence intervals observed for these markers—particularly vitamin B12 (95% CI: 1.000–1.002) and HDL (95% CI: 0.61–4.30)—indicate considerable uncertainty, and it remains plausible that larger, multi-center studies with greater statistical power could detect modest independent effects. Therefore, our findings should be interpreted as suggesting that these markers did not demonstrate independent associations in this specific cohort, rather than as a categorical refutation of their potential predictive value.

The number of prior vascular interventions emerged as the strongest independent predictor of subsequent thrombosis in our model. This likely reflects the cumulative burden of underlying pathologic processes—such as aggressive neointimal hyperplasia and vessel wall remodeling—rather than iatrogenic injury per se. Indeed, each intervention is typically performed to rescue a failing fistula, and repeated procedures signal progressive vascular disease (21). These findings underscore the clinical importance of preserving native vessel integrity through optimal cannulation practices, early detection of stenosis, and judicious use of interventions. Strategies aimed at mitigating the biological drivers of venous stenosis may ultimately prove more effective in preventing thrombosis than reactive procedural approaches. Hypertension was also confirmed as a powerful independent risk factor for AVF thrombosis, with an hazard ratio of 2.24(95% CI: 0.92–5.43)in the adjusted model. Elevated systemic blood pressure increases intraluminal pressure, wall shear stress, and endothelial damage, all of which accelerate the development of stenosis and thrombotic occlusion (22). Moreover, hypertension is a potentially modifiable factor, and our findings underscore the importance of strict blood pressure control in preserving vascular access patency. Routine blood pressure monitoring and optimization of antihypertensive therapy should be integral components of vascular access management in hemodialysis patients. Studies have shown that hypertension may affect the patency rate of arteriovenous fistulas through mechanisms such as promoting neointimal hyperplasia (NIH) and atherosclerosis (23). In one study, NIH was identified as the primary cause of AVF dysfunction, and different morphological types of lesions were closely associated with patency rates following percutaneous transluminal angioplasty (24). This suggests that hypertension may reduce primary and secondary patency rates of AVF by exacerbating the formation of NIH.

Notably, diabetes was associated with a lower risk of AVF thrombosis in the adjusted model (HR = 0.63, *p* = 0.091). However, this result should be interpreted with considerable caution. Current evidence regarding the association between diabetes and AVF thrombosis remains inconsistent, with numerous clinical studies reporting no significant independent relationship between diabetes and AVF dysfunction or occlusion (25). Potential reasons for this unexpected finding include residual confounding, unmeasured differences in baseline vascular status, or the relatively small number of thrombotic events in the present cohort. Given the inconsistent findings across the literature, we cannot conclude that diabetes exerts a genuine protective effect. Rather, this observation underscores the complex and non-uniform relationship between diabetes and AVF outcomes, which requires further verification in large-scale prospective studies.

Our study has several notable strengths. First, we explicitly adjusted for surgical frequency, a key mechanical variable rarely addressed in nutrition-focused studies. Second, we used multiple imputation to reduce bias from missing data. Third, we comprehensively evaluated model performance using discrimination (AUC 0.781, 95% CI 0.735–0.827), calibration (Hosmer-Lemeshow *p* = 0.591), and decision curve analysis, supporting of our findings. Finally, the large sample size enhances statistical power and generalizability within similar clinical settings.

Several limitations should also be acknowledged. This was a retrospective, single-center study, which may limit external generalizability. Residual confounding from unmeasured factors, such as specific medications, coagulation function, or dialysis adequacy, cannot be excluded. Although the proportional hazards assumption held for all core predictors of interest, the global test showed a borderline *p*-value (*p* = 0.032), primarily driven by age and gender. Nevertheless, the consistency across multiple sensitivity analyses supports the robustness of our main conclusions. The high proportions of missing values in several variables (e.g., vitamin B12, 59.5%) necessitated multiple imputation. In sensitivity analyses, complete-case analysis (*n* = 165) yielded a higher AUC (0.866) and showed variations in the statistical significance of some predictors compared with the primary model. These discrepancies are likely due to the substantially reduced sample size and potential selection bias in the complete-case subset, whereas multiple imputation preserved all 783 patients and provided more stable estimates under the missing at random assumption. Importantly, the direction of the associations for surgery count, hypertension, and diabetes remained consistent across all analytical approaches, supporting the robustness of the key conclusions. In addition, the unexpected association between diabetes and lower thrombosis risk requires further investigation in prospective, multi-center cohorts. The potential for confounding by indication and reverse causation must be acknowledged. The number of prior surgical procedures may reflect the severity of underlying vascular pathology rather than a purely causal exposure, as procedures are typically performed in response to existing dysfunction (e.g., stenosis or reduced flow). Consequently, patients with more severe vascular disease receive more interventions and are simultaneously at higher risk for subsequent thrombosis. Although we adjusted for available confounders, residual confounding from unmeasured vascular pathology (e.g., extent of neointimal hyperplasia, vessel geometry, or flow characteristics) cannot be excluded. Therefore, we emphasize that surgery count should be interpreted as a composite risk marker rather than a direct causal exposure, and our findings primarily support enhanced surveillance and risk stratification rather than a blanket recommendation to avoid necessary procedures. Although the EPV ratio for the final Cox model (19.2) exceeded the recommended minimum of 10, we acknowledge that the initial screening step involved 11 candidate variables. However, bootstrap internal validation demonstrated excellent model stability with minimal optimism (0.0065), suggesting that overfitting is unlikely to be a major concern. Although our study had adequate power to detect the dominant effect of surgical burden (HR = 1.44), it was not specifically powered to detect modest independent effects of nutritional-metabolic indicators. The wide confidence intervals for vitamin B12, HDL, and folate suggest that smaller but potentially clinically meaningful associations cannot be excluded. Future larger-scale, multi-center studies are warranted to more definitively address the role of nutritional status in AVF thrombosis. Several important clinical confounders were not available in our retrospective dataset, including dialysis vintage, access flow measurements, artery diameter, previous access failure history, stenosis history, cannulation technique, antiplatelet or anticoagulant use, erythropoietin use, dialysis adequacy (Kt/V), ultrafiltration burden, infection history, and detailed vascular access surveillance data. Although we adjusted for available confounders including surgery count, hypertension, diabetes, age, gender, and fistula diameter, residual confounding from these unmeasured factors cannot be excluded. Future prospective studies incorporating these variables are warranted to more definitively assess the independent role of nutritional status in AVF thrombosis.

## 5 Conclusion

After adjusting for surgical frequency and clinical confounders, conventional nutritional-metabolic indicators did not demonstrate independent associations with AVF thrombosis in this single-center cohort. Cumulative surgical burden and hypertension emerged as the predominant independent risk markers in this analysis. However, the wide confidence intervals for nutritional-metabolic indicators suggest that modest effects cannot be excluded, and larger multi-center studies are needed to more definitively assess the role of nutritional status in AVF thrombosis. Our findings should be interpreted within the context of the study‘s observational design and limited power to detect smaller associations. These findings suggest that nutritional biomarkers should be interpreted cautiously in clinical risk stratification. Notably, surgery count may reflect underlying vascular disease severity rather than purely causal exposure, and should be understood as a composite risk marker. Future research and clinical practice should prioritize early identification of patients with high cumulative surgical burden and implement intensified surveillance strategies, while acknowledging that many procedures are clinically necessary responses to progressive vascular pathology.

## Data Availability

The raw data supporting the conclusions of this article will be made available by the authors, without undue reservation.
