# Supplementary material for: Nutritional-metabolic indicators do not independently predict AVF thrombosis after adjustment for surgical frequency: a retrospective cohort study
Source: Front Nutr. 2026 Jul 27;13:1845230. doi: 10.3389/fnut.2026.1845230 (PMC13453830; doi:10.3389/fnut.2026.1845230)
Supplement: Supplementary file 1 [file Table_1.DOCX]

**Supplementary Table S1. Comparison of baseline characteristics between included and excluded patients**

| Variable | Included (n = 783) | Excluded (n = 183) | P |
| --- | --- | --- | --- |
| Age (years), mean ± SD | 60.18 ± 13.08 | 61.33 ± 12.74 | 0.303 |
| Gender (Male), n (%) | 534 (68.2) | 121 (66.9) | 0.793 |
| Hypertension, n (%) | 693 (88.5) | 168 (91.8) | 0.247 |
| Diabetes, n (%) | 518 (66.2) | 119 (65.0) | 0.839 |
| Surgery count, mean ± SD | 0.98 ± 1.18 | 1.19 ± 0.51 | 0.409 |
| Albumin (g/L), mean ± SD | 66.14 ± 9.31 | 65.22 ± 10.89 | 0.275 |
| Hemoglobin (g/L), mean ± SD | 93.21 ± 27.42 | 92.04 ± 23.81 | 0.619 |
| BMI (kg/m²), mean ± SD | 23.45 ± 3.79 | 23.68 ± 3.77 | 0.589 |

This table presents the complete-case sensitivity analysis (n = 165). Results should be interpreted with caution due to the substantially reduced sample size and potential selection bias. The primary predictors (surgery count and hypertension) remained consistent with the main imputed model (Table 4).
