# Supplementary material for: Nutritional-metabolic indicators do not independently predict AVF thrombosis after adjustment for surgical frequency: a retrospective cohort study
Source: Front Nutr. 2026 Jul 27;13:1845230. doi: 10.3389/fnut.2026.1845230 (PMC13453830; doi:10.3389/fnut.2026.1845230)
Supplement: Supplementary file 2 [file Table_2.DOCX]

### Supplementary Table S2. Association between missingness and AVF thrombosis

| Variable | Missing % | OR for missingness | 95% CI | P |
| --- | --- | --- | --- | --- |
| Vitamin B12 | 59.5 | 1.08 | 0.72–1.62 | 0.482 |
| Folate | 56.7 | 0.95 | 0.63–1.43 | 0.734 |
| hs-CRP | 42.3 | 1.12 | 0.74–1.70 | 0.283 |
| Surgery count | 39.1 | 0.88 | 0.58–1.33 | 0.521 |
| Ferritin | 38.3 | 1.05 | 0.69–1.59 | 0.671 |
| LDL | 34.2 | 0.92 | 0.61–1.38 | 0.615 |
| HDL | 34.0 | 1.10 | 0.73–1.66 | 0.394 |

OR > 1 indicates that patients with thrombosis were more likely to have missing values for that variable.
