# Supplementary material for: Nutritional-metabolic indicators do not independently predict AVF thrombosis after adjustment for surgical frequency: a retrospective cohort study
Source: Front Nutr. 2026 Jul 27;13:1845230. doi: 10.3389/fnut.2026.1845230 (PMC13453830; doi:10.3389/fnut.2026.1845230)
Supplement: Supplementary file 3 [file Table_3.DOCX]

### Supplementary Table S3. Missing indicator method: association between missingness indicators and AVF thrombosis

| Missing indicator | OR | 95% CI | P |
| --- | --- | --- | --- |
| hs_crp_miss | 1.05 | 0.80–1.38 | 0.717 |
| d_dimer_miss | 0.92 | 0.74–1.13 | 0.427 |
| hdl_miss | 1.01 | 0.82–1.25 | 0.982 |
| ldl_miss | 1.01 | 0.82–1.25 | 0.982 |
| vitb12_miss | 1.07 | 0.85–1.35 | 0.564 |
| folate_miss | 1.06 | 0.86–1.31 | 0.456 |
| surgery_count_miss | 0.82 | 0.65–1.03 | 0.092 |
