# Supplementary material for: Nutritional-metabolic indicators do not independently predict AVF thrombosis after adjustment for surgical frequency: a retrospective cohort study
Source: Front Nutr. 2026 Jul 27;13:1845230. doi: 10.3389/fnut.2026.1845230 (PMC13453830; doi:10.3389/fnut.2026.1845230)
Supplement: Supplementary file 4 [file Table_4.DOCX]

**Supplementary Table S4. Internal validation of predictive models (bootstrap, 500 resamples)**

| Model | Metric | Apparent | Bootstrap mean | Optimism | Optimism-corrected |
| --- | --- | --- | --- | --- | --- |
| Logistic regression | AUC | 0.845 | 0.850 | 0.0053 | 0.840 |
| Logistic regression | Calibration slope | 6.132 | 6.189 | 0.057 | 6.057 |
| Cox regression | C-index | 0.740 | 0.746 | 0.0065 | 0.733 |
