# Supplementary material for: Nutritional-metabolic indicators do not independently predict AVF thrombosis after adjustment for surgical frequency: a retrospective cohort study
Source: Front Nutr. 2026 Jul 27;13:1845230. doi: 10.3389/fnut.2026.1845230 (PMC13453830; doi:10.3389/fnut.2026.1845230)
Supplement: Supplementary file 5 [file Table_5.DOCX]

Supplementary Table S5. Comparison of baseline characteristics between patients with observed vs. missing values for high-missing variables

| Variable | Missing % | Age | Albumin | Hemoglobin | BMI | Gender | Hypertension | Diabetes |
| --- | --- | --- | --- | --- | --- | --- | --- | --- |
| Vitamin B12 | 59.5% | 0.312 | 0.036 | 0.123 | 0.199 | 0.907 | 1.000 | 1.000 |
| Folate | 56.7% | 0.420 | 0.009 | 0.404 | 0.178 | 1.000 | 0.989 | 0.328 |
| hs-CRP | 42.3% | 0.604 | 0.029 | 0.773 | 0.564 | 0.580 | 0.669 | 0.602 |
| Surgery count | 39.1% | 0.888 | 0.865 | 0.465 | 0.848 | 0.394 | 0.331 | 0.863 |
| HDL | 34.0% | 0.263 | <0.001 | 0.849 | 0.001 | 0.884 | 0.118 | 0.004 |
| LDL | 34.2% | 0.340 | <0.001 | 0.862 | 0.002 | 0.958 | 0.108 | 0.003 |

Values are P-values comparing observed vs. missing groups for each variable. P-values were calculated using the Mann-Whitney U test for continuous variables (age, albumin, hemoglobin, BMI) and the chi-square test for categorical variables (gender, hypertension, diabetes).
