# Supplementary material for: Nutritional-metabolic indicators do not independently predict AVF thrombosis after adjustment for surgical frequency: a retrospective cohort study
Source: Front Nutr. 2026 Jul 27;13:1845230. doi: 10.3389/fnut.2026.1845230 (PMC13453830; doi:10.3389/fnut.2026.1845230)
Supplement: Supplementary file 6 [file Table_6.DOCX]

**Supplementary Table S6. Cox regression analysis with surgery count as categorical variable**

| Surgery count | HR (95% CI) | P |
| --- | --- | --- |
| 0 | 1.00 (Reference) | — |
| 1 | 5.47 (2.39–12.49) | < 0.001 |
| 2 | 8.71 (3.77–20.14) | < 0.001 |
| ≥3 | 13.06 (4.61–36.96) | < 0.001 |
| P for trend |  | < 0.001 |

Adjusted for hypertension, diabetes, age, and gender.
